# Evaluation of Serum Fructosamine as Diagnostic Marker of Postoperative Recurrence in the Patients with Breast Cancer

**DOI:** 10.1155/2023/6435776

**Published:** 2023-07-12

**Authors:** Yuanqing Zhao, Xuelong Xie, Jingling Xie, Limei Zhang, Baolin Li, Jinbo Liu, Hui Jiang

**Affiliations:** ^1^Department of Laboratory Medicine, The Affiliated Hospital of Southwest Medical University, Sichuan Province Engineering Technology Research Center of Molecular Diagnosis of Clinical Diseases, Molecular Diagnosis of Clinical Diseases Key Laboratory of Luzhou, Sichuan 646000, China; ^2^Department of Laboratory Medicine, The Second People's Hospital of Yibin, Sichuan 644000, China

## Abstract

**Objectives:**

A series of laboratory parameters were screened to identify the proper serum markers that could be used to predict breast cancer recurrence at an early stage.

**Methods:**

A case-control retrospective study on 224 patients without postoperative recurrence and 43 patients with postoperative recurrence of breast cancer was performed. The edgeR software package was used to identify the test indicators expressed differently between the two groups. Univariate analysis was used to screen for diagnostic marker that could predict postoperative recurrence of breast cancer. In addition, the differential test indicators at different time points from surgery to recurrence were collected in patients with postoperative recurrence of breast cancer as a verification database.

**Results:**

We screened out three test indicators (TBA, GSP, and URBC) for differential expression, which were all expressed downregulated in the postoperative recurrence group of breast cancer. Univariate analysis suggested that only the difference in GSP levels between the two groups was statistically significant (*P* = 0.001). ROC curve analysis showed that the area under the curve of GSP was 0.662, while the area under the curve of GSP+AFP+CEA+CA125+CA153+age was increased to 0.828. In addition, serum GSP levels were significantly reduced after recurrence compared with before recurrence in breast cancer patients (*P* < 0.01).

**Conclusions:**

In summary, GSP could be used for early diagnosis of breast cancer recurrence after surgery, and the predicted value of combining GSP, tumor markers, and age was better than that of individual indicators.

## 1. Introduction

Breast cancer, one of the most common malignancies in women, poses a serious threat to human health and is the second leading cause of death from cancer in women [1]. In China, there are about 304,000 new confirmed cases of breast cancer and 70,000 deaths each year [2]. And the incidence of breast cancer is also increasing year by year, especially in coastal areas or economically developed areas, where the incidence of breast cancer ranks highest [3]. The choice of treatment for breast cancer depends on the pathological type, stage, sensitivity to hormones, age of the patient, and overall health status. According to the data from the American Institute for Cancer Research in 2015, the main treatment modalities for breast cancer include surgery, chemotherapy, biological therapy, targeted therapy, hormone therapy, and chemotherapy. Among them, surgical resection is the most important treatment strategy for breast cancer [4]. However, some patients may still have small residual lesions after surgery, which is a key cause of distant recurrent metastases. As a result, a significant proportion of patients experience tumor recurrence after surgery, and the probability of recurrence (10% to 41%) depends on the grading stage of the tumor, which greatly affects the patient's survival prognosis [5]. Therefore, early recognition of postoperative recurrence of breast cancer is of great clinical significance for improving breast cancer survival.

Currently, postoperative recurrence monitoring of breast cancer relies heavily on routine mammographic screening. For screen-positive patients, a histopathological biopsy is then performed [6]. However, these tests may lead to overtesting, and the cost of testing is high, which can easily cause anxiety in patients. For stage II colon cancer, postoperative CEA is a strong predictor of cancer recurrence [7–9]. Because serum markers have the advantages of low invasiveness, low cost, and easy detection, serum marker testing is the most suitable method for routine clinical screening. However, there is currently no effective serum biomarker that can be used for the early diagnosis of breast cancer relapse [10]. Therefore, it is significant to find effective serum markers with high sensitivity and specificity for postoperative prediction of breast cancer [11–13]. In this study, we intended to screen out serum markers with predictive ability for postoperative recurrence from a series of laboratory parameters, which are routine test items of breast cancer after surgery, thereby reducing the probability of postoperative recurrence of breast cancer patients and improving their survival prognosis.

## 2. Materials and Methods

### 2.1. Patients

A case-control retrospective study on 267 breast cancer patients was performed. Breast cancer patients after surgery diagnosed and treated at the Affiliated Hospital of Southwest Medical University from January 2020 to April 2021 were selected as research subjects. Inclusion criteria were as follows: (1) met the diagnostic criteria for breast cancer in the “Guidelines and Specifications for the Diagnosis and Treatment of Breast Cancer of the Chinese Anti-Cancer Association (2017 Edition)” and confirmed by pathological and immunohistochemical testing and (2) after breast-conserving surgery or breast cancer radical resection. Exclusion criteria were as follows: (1) coexisting other malignant tumors and (2) recurrence after multiple surgeries. According to the inclusion and exclusion criteria, a total of 267 breast cancer patients were enrolled in this study, which were divided into recurrence group (*n* = 43) and a nonrecurrence group (*n* = 224) according to postoperative recurrence. The recurrence group accounted for 78.6% of patients aged >50 years, while the nonrecurrence group accounted for 52.9% of patients aged >50 years.

### 2.2. Information Collection

Postoperative medical records of breast cancer patients were collected, including age, sex, WHO grade, TNM stage, molecular classification, pathological type, immunohistochemistry, surgical methods (including modified radical resection and breast conservation), and postoperative adjuvant therapy. Laboratory indicators (Supplementary file (available here)) were collected for two groups of breast cancer patients (postoperative recurrence and nonrecurrence). In addition, the trend of differential test indicators in patients with postoperative recurrence of breast cancer from postoperative to recurrence was analyzed.

### 2.3. Screening of Test Indicators for Differential Expression

The Bioconductor R software package was used to filter and fill in the gaps. The criteria for the inclusion of test data were standard deviation > 0.01, mean > 0.05, and proportion of null values < 25%. The *k*-nearest neighbour method was used to fill in the gaps. The edgeR software package was used to standardize the data, and then, the differential expression index was screened out by comparing the two groups. The screening criteria were false discovery rate (FDR) < 0.05 and |log2FC| > 1.

### 2.4. Evaluation of the Diagnostic Value of the Differential Expression Index

Univariate analysis was applied to find diagnostic marker of postoperative recurrence. The receiver operating characteristic (ROC) curve was plotted by the survivalROC software package, and the area under the curve (AUC) was calculated to assess the diagnostic value of GSP expression level on postoperative recurrence in breast cancer patients.

### 2.5. Statistical Analyses

Data analysis and plotting were completed by SPSS 23.0 statistics software and GraphPad Prism 8.0 software. The enumeration data was expressed by frequency number (percentage), and the chi-square test was used for intergroup comparison. The measurement data with normal distribution were compared between the two groups using independent sample *t*-test. The measurement data of the skewed distribution were compared between the two groups using the Mann–Whitney nonparametric test of two independent samples. Binary logistic regression analysis was applied to construct a joint index. *P* < 0.05 was considered that the difference was statistically significant.

### 2.6. Ethical Approval

This study was approved by the Institutional Review Board of the Affiliated Hospital of Southwest Medical University (KY2020043) in accordance with the Declaration of Helsinki.

### 2.7. Informed Consent

The Institutional Review Board of the Affiliated Hospital of Southwest Medical University waived the requirement of informed consent as this study was a retrospective analysis.

## 3. Results

### 3.1. Identification of Differential Test Indicators

**Figure 1:**
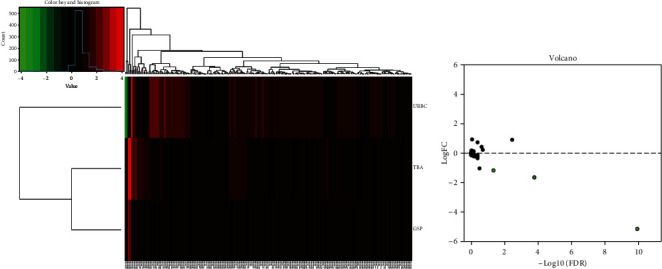
Differentially expressed indicators between the two groups of breast cancer patients. (a) Clustering heat map. (b) Volcano plot.

In order to screen out the differential test indicators between patients with postoperative recurrence (*n* = 43) and nonrecurrence (*n* = 224), we analyzed the differences of expression of a series of test indicators (urine routine, coagulation test, biochemical I+electrolyte 5, blood count, and thymidine kinase 1) between the two groups. Three downregulated indicators (TBA, GSP, and URBC) were identified (Table 1). The clustering heat map of difference indices is shown in Figure 1(a), and the volcano map is shown in Figure 1(b).

### 3.2. Evaluation of the Diagnostic Value of the Differential Test Indicators

**Figure 2:**
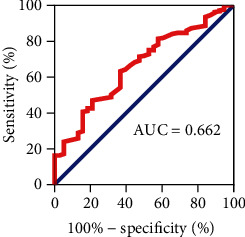
Diagnostic accuracy of GSP for postoperative recurrence of breast cancer assessed by the AUC.

In this study, we found that the GSP level of 38 patients with postoperative recurrence of breast cancer was 2.0 ± 0.2 (mmol/L). The GSP level of 221 patients who did not relapse after breast cancer surgery was 2.1 ± 0.3 (mmol/L). The difference in GSP levels between the two groups was statistically significant (*t* = 3.224, *P* = 0.001) (Table 2). Although the previous results of the R language analysis showed that the levels of TBA and ULBC were significantly different between the two groups, the differences disappeared when using SPSS software for univariate analysis. The reason might be that the two calculation methods were different. However, no matter which analysis method was used, the GSP expression was always different, which also verified the reliability of the results from another aspect. The ROC curve was then used to assess the sensitivity and specificity of postoperative recurrence prediction of breast cancer. As shown in Figure 2, the AUC area of GSP was 0.662, indicating that GSP had good predictive ability.

### 3.3. Validation of Differentially Expressed Serum Indicators

**Figure 3:**
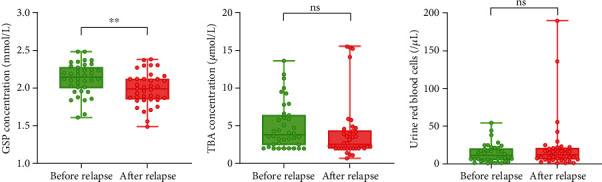
Validation of differentially expressed serum indicators. The serum GSP (a), serum TBA (b), and urine RBC (c) levels in 25 patients with postoperative recurrence of breast cancer at two time points after recurrence and half a year before recurrence were collected and compared (^∗∗^*P* < 0.01).

In order to further verify the reliability of the serum indicator GSP as a marker for predicting postoperative recurrence, we collected serum TBA, serum GSP, and urine RBC indicators in the patients with postoperative recurrence of breast cancer (*n* = 38) half a year before recurrence. The index changes of these patients at different time points were compared. The results showed that the serum GSP level was significantly reduced after the patient's recurrence (*P* < 0.01) (Figure 3(a)), while there was no significant change in the other 2 indicators (Figures 3(b) and 3(c)). The above results suggested that serum GSP was a promising marker for predicting postoperative recurrence.

### 3.4. Expression of Tumor Markers in the Recurrence Group and the Nonrecurrence Group after Breast Cancer Surgery

As shown in the table, CA153 was expressed at a higher level in the postoperative recurrence group of breast cancer than in the nonrecurrence group, with a statistically significant difference (*P* < 0.05). There were no significant differences in the expression of AFP, CEA, CA125, and CA199 between the two groups (Table 3).

### 3.5. The Diagnostic Value of Joint Index in Predicting Postoperative Recurrence of Breast Cancer

In Table 3, although the differences of AFP, CEA, CA125, and CA199 were not statistically significant, they were clinically considered as independent variables closely related to the dependent variables. Therefore, GSP, AFP, CEA, CA125, CA153, CA199, and age were included in the binary logistic regression analysis, and the results showed that GSP, AFP, CEA, CA125, CA153, and age were independent variables in the diagnostic model for early diagnosis of postoperative recurrence.

**Figure 4:**
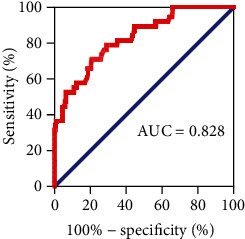
Diagnostic accuracy of the joint index (GSP+AFP+CEA+CA125+CA153+age) for postoperative recurrence of breast cancer assessed by the AUC.

The joint factor GSP+AFP+CEA+CA125+CA153+age was constructed by binary logistic regression analysis. The probability value of the joint index was used to represent the detection level of the joint index. The ROC curve of the joint index was plotted, and the AUC area was calculated. The results of ROC curve analysis showed that the AUC of the joint indicator was 0.828, which was greater than that of individual indicators (Figure 4). In addition, the best diagnostic thresholds for GSP, AFP, CEA, CA125, and CA153 were 2.045 (mmol/L), 2.435 (ng/mL), 2.545 (ng/mL), 17.7 (U/mL), and 17.25 (U/mL), respectively (Table 4). It was suggested that the diagnostic value of combining GSP, serum tumor markers, and age in predicting postoperative recurrence of breast cancer was higher than that of individual indicator GSP, and the combined indicator had an excellent ability to predict postoperative recurrence.

## 4. Discussion

In recent years, with the increase of women's life and work pressure, the incidence of breast cancer has also been rising, and it is expanding toward a younger group [14, 15]. At present, radical surgery is the preferred solution for the treatment of breast cancer [16, 17]. However, breast cancer is a systemic disease. Some patients have experienced local tumor cell metastasis and proliferation at the time of treatment, so the survival time of surgical treatment alone is not ideal. Postoperative micrometastasis foci initiate rapid growth mode, and early adjuvant chemotherapy can kill tiny metastatic cancers and residual tumor cells as soon as possible, thereby reducing the recurrence rate and improving survival [18–20]. Therefore, predicting the recurrence trend of tumors as early as possible and treating them in a timely manner can significantly improve the prognosis and survival rate of breast cancer patients.

Some clinical features of patients are considered risk factors for breast cancer, such as age, family history, reproductive factors, estrogen, and lifestyle [21–23]. Among them, age has the greatest impact on the occurrence of breast cancer, which is at the bottom of pyramid of the breast cancer risk factor [24]. In 2013, among the new breast cancer cases in the United States, 12,880 were under the age of 40, 51,680 were 40-49 years old, 110,980 were 50-64 years old, and 121,440 were over the age of 65. Similarly, the number of breast cancer-related deaths increased with age [25]. Among the patients with postoperative recurrence of breast cancer included in this study, 21.4% were ≤50 years old and 78.6% were >50 years old, suggesting that age also significantly affected the postoperative recurrence rate of breast cancer. Because the data on breast cancer pathology, WHO grade, and cancer stage of some patients collected in this study were incomplete, we did not analyze the effects of these factors on postoperative recurrence of breast cancer. In future studies, we will expand the sample size, enrich the research factors, and conduct multicenter studies to further explore the risk factors for breast cancer recurrence after surgery.

In addition, a number of serum tumor markers also contribute to the early diagnosis of breast cancer, including AFP, CEA, CA125, CA153, and CA199. A paper on diagnostic markers for metastatic breast cancer [26] reported that the sensitivity and specificity of CEA and CA125 for the diagnosis of metastatic breast cancer alone were 56.7% and 97.0%, respectively. The sensitivity of CA153 and CA125 in combination in the diagnosis of metastatic breast cancer was 91.5%. The sensitivity and specificity of CEA and CA153 in the diagnosis of bone metastases were 77.1% and 45.8%, respectively. In this study, CA153 level was significantly different in the postoperative recurrence group and the nonrecurrence group of breast cancer. In addition, the multivariate analysis results showed that the levels of CEA, CA125, and CA153 were influencing factors for postoperative recurrence of breast cancer. Although there were no statistically significant differences between AFP, CEA, CA125, and CA199 in the univariate analysis, it was clinically believed that these indicators were also possible risk factors for breast cancer recurrence. Therefore, these indicators were included as independent variables in the multivariate analysis. These results demonstrated the potential of serum tumor markers for predicting postoperative recurrence of breast cancer.

GSP is a substance formed by proteins in plasma during the nonenzymatic glycation of glucose. Since the half-life of plasma proteins is 17 days, GSP reflects the blood glucose level within 2-3 weeks, and the amount of its formation depends on the blood glucose concentration. In the study, Wulaningsih et al. [27] observed a strong positive correlation between blood glucose levels and cancer risk, while there was a negative correlation between GSP levels and cancer risk. Similar results could be observed in prostate, lung, and colon cancers. As we all know, glucose metabolism has always been the core of the field of cancer metabolism, and GSP is an important marker that reflects the metabolism of glucose in the human body. This could be used to explain why blood glucose and GSP levels were closely linked to the occurrence and development of tumors. Our findings also found that GSP was an independent predictor of postoperative recurrence of breast cancer. The above results suggested that GSP was expected to become a potential marker of tumor diagnosis and prognosis.

However, the sensitivity and specificity of GSP in predicting postoperative recurrence of breast cancer were only 63.3% and 63.2%. In order to further address the limitations of GSP in the prediction of postoperative recurrence of breast cancer, we combined GSP with serum tumor markers and age. In this study, the sensitivity and specificity of the prediction of the joint index increased to 71.1% and 80.0%, and the AUC area increased from 0.662 to 0.828. It showed that the prediction ability of the joint index was greatly improved, and the joint index had potential value in the early prediction of breast cancer recurrence after surgery.

In summary, GSP could be used for the early diagnosis of breast cancer recurrence after surgery. The combination of GSP and tumor markers and age was better predictive than that of individual indicators. The results of this study improve the diagnostic efficacy of small residual lesions after breast cancer surgery, which could be used as an auxiliary examination to provide more reference information and diagnostic evidence for clinical practice.

## Figures and Tables

**Table 1:** The difference test index between the two groups of breast cancer patients.

Index	logFC	logCPM	*P* value	FDR
TBA	-5.14*E*+00	1.51*E*+01	1.93*E*-12	1.18*E*-10
GSP	-1.65*E*+00	1.12*E*+01	5.57*E*-06	1.70*E*-04
URBC	-1.17*E*+00	1.36*E*+01	3.12*E*-03	4.76*E*-02

TBA: total bile acid; GSP: fructosamine; URBC: urine red blood cells; FC: fold change; CPM: counts per million; FDR: false discovery rate.

**Table 2:** Comparison of age and the three test indicators between the recurrence group and nonrecurrence group by univariate analysis.

	Recurrence group (*n* = 38^a^)	Nonrecurrence group (*n* = 221^a^)	*x* ^2^/*t*	*P*
Age^b^ (Y), *n* (%)				
≤50	8 (21.4)	104 (47.1)	8.935	0.003
>50	30 (78.6)	117 (52.9)		
TBA (*μ*mol/L)	4.1 ± 4.0	4.7 ± 5.1	0.637	0.525
GSP (mmol/L)	2.0 ± 0.2	2.1 ± 0.3	3.224	0.001
URBC (/*μ*L)	21.8 ± 36.1	18.3 ± 30.4	-0.624	0.533

^a^Partial information of eight patients was missing, so the number of cases used for subsequent univariate analysis was 259. ^b^Age referred to the age at which the patient was first diagnosed with breast cancer.

**Table 3:** Comparison of serum tumor marker levels between the recurrence group and the nonrecurrence group by univariate analysis.

	Recurrence group (*n* = 38)	Nonrecurrence group (*n* = 221)	*t*	*P*
AFP (ng/mL)	22.5 ± 114.3	3.6 ± 4.1	-1.020	0.314
CEA (ng/mL)	7.4 ± 17.9	1.6 ± 2.9	-2.003	0.052
CA125 (U/mL)	36.6 ± 88.5	11.1 ± 7.9	-1.776	0.084
CA153 (U/mL)	26.2 ± 38.0	10.9 ± 5.3	-2.464	0.018
CA199 (U/mL)	14.7 ± 16.6	20.2 ± 113.1	0.300	0.764

**Table 4:** ROC curve analysis of different indexes for diagnosis of postoperative recurrence of breast cancer.

Index	AUC	*P*	95% CI	Cutoff	Youden index	Sensitivity	Specificity
GSP	0.662	0.001	0.573-0.750	2.045 (mmol/L)	0.265	63.3%	63.2%
AFP	0.535	0.486	0.434-0.637	2.435 (ng/mL)	0.121	76.3%	35.7%
CEA	0.656	0.002	0.553-0.760	2.545 (ng/mL)	0.294	42.1%	87.3%
CA125	0.615	0.023	0.509-0.721	17.7 (U/mL)	0.286	39.5%	89.1%
CA153	0.637	0.007	0.527-0.747	17.25 (U/mL)	0.294	42.1%	87.3%
Joint index	0.828	0.000	0.758-0.898	—	0.507	71.1%	80.0%

## Data Availability

The datasets generated during and/or analyzed during the current study are available from the corresponding authors on reasonable request.

## Supplementary Materials

Supplementary 1 The detailed information of routine test items of breast cancer after surgery.

Supplementary 2 STROBE statement—checklist of items that should be included in reports of case-control studies.
