# Supplementary material for: Evaluation of Serum Fructosamine as Diagnostic Marker of Postoperative Recurrence in the Patients with Breast Cancer
Source: Biomed Res Int. 2023 Jul 12;2023:6435776. doi: 10.1155/2023/6435776 (PMC10356523; doi:10.1155/2023/6435776)
Supplement: Supplementary 1 — The detailed information of routine test items of breast cancer after surgery. [file 6435776.f1.docx]

**Supplementary file**

**Evaluation of serum fructosamine as diagnostic marker of postoperative recurrence in the patients with breast cancer**

Yuanqing Zhao^1^, Xuelong Xie^1,2^, Jingling Xie^1^, Limei Zhang^1^, Baolin Li^1^, Jinbo Liu^1^*, Hui Jiang^1^*

^1^Department of Laboratory Medicine, The Affiliated Hospital of Southwest Medical University, Sichuan Province Engineering Technology Research Center of Molecular Diagnosis of Clinical Diseases, Molecular Diagnosis of Clinical Diseases Key Laboratory of Luzhou, 646000, China

^2^Department of Laboratory Medicine, Second People's Hospital of Yibin, Yibin, 644000, China

*Correspondence:

*Hui Jiang and Jinbo Liu are co-corresponding authors.

Dr Jinbo Liu, Department of Laboratory Medicine, The Affiliated Hospital of Southwest Medical University, 25 Taiping Street, Luzhou, Sichuan 646000, P.R. China.

Email: liulab2019@163.com (JB. Liu)

**Routine test items of breast cancer after surgery include:**

1. **Urine routine:** urine PH value (PH), urine specific gravity (SG), urine white blood cells (UWBC), urine red blood cells (URBC), epithelial cells (EC), bacteria (BACT), small round cells (SRC), crystal (XTAL), yeast like bacteria (YLC), tube type (CAST), pathological tube type (P.CAST), mucus filament (MUCUS), conductivity (Cond);
2. **Coagulation test:** prothrombin time (PT), international standardized ratio (PT-INR), prothrombin time ratio (PT-RATIO), prothrombin time activity (PT -%), activated partial thrombin time (APTT), thrombin time (TT), fibrinogen (Fib);
3. **Biochemical I + electrolytes 5:** alanine aminotransferase (ALT), aspartic aminotransferase (AST), aspartic aminotransferase/alanine aminotransferase (AST/ALT), total protein (TP), albumin (ALB), globulin (GLO), albumin/globulin (A/G), total bilirubin (TBIL), direct bilirubin (DBIL), indirect bilirubin (IBIL), total bile acid (TBA), lactate dehydrogenase (LDH), r-glutamyltransferase (GGT), alkaline phosphatase (ALP), prealbumin (PA), urea (Urea), uric acid (UA), creatinine (Crea), total cholesterol (TC), triglyceride (TG), high density lipoprotein cholesterol (HDL-C), low density lipoprotein cholesterol (LDL-C), apolipoprotein A1 (APOA1), apolipoprotein B (APOB), glucose (GLU), fructosamine (GSP), potassium (K), sodium (Na), chlorine (Cl), calcium (Ca), carbon dioxide (CO2), retinol binding protein (RBP), glomerular filtration rate (GFR), hemolysis （Hemo), jaundice (Icte), lipoid turbidity (Lipe), anion gap (AG);
4. **Blood routine examination:** white blood cell count (WBC), neutrophil count (NEU), lymphocyte count (LYM), monocyte count (MONO), eosinophils count (EOS), basophil count (BASO), neutrophil rate (NEU-R), lymphocyte rate (LYM-R), monocyte rate (MONO-R), eosinophils rate (EOS-R), basophil rate (BASO-R), red blood cell count (RBC), hemoglobin (HGB), hematocrit (HCT), mean corpuscular volume (MCV), mean corpuscular hemoglobin (MCH), mean corpuscular hemoglobin concentration (MCHC), standard deviation of red cell volume distribution width (RDW-SD), coefficient of variation of red cell volume distribution width (RDW-CV), platelet count (PLT), mean platelet volume (MPV), platelet hematocrit (PCT), platelet volume distribution width (PDW), and large platelet ratio (P-LCR);
5. Thymidine kinase 1;
6. **Carcinoembryonic antigen:** alpha fetoprotein (AFP), carcinoembryonic antigen (CEA), carbohydrate antigen 125 (CA125), carbohydrate antigen 153 (CA153), carbohydrate antigen 199 (CA199).
